# Impact of smoking amount on clinicopathological features and survival in non-small cell lung cancer

**DOI:** 10.1186/s12885-020-07358-3

**Published:** 2020-09-03

**Authors:** Woo Ho Ban, Chang Dong Yeo, Solji Han, Hye Seon Kang, Chan Kwon Park, Ju Sang Kim, Jin Woo Kim, Seung Joon Kim, Sang Haak Lee, Sung Kyoung Kim

**Affiliations:** 1 Division of Pulmonary, Critical Care and Sleep Medicine, Department of Internal Medicine, Eunpyeong St. Mary’s Hospital, College of Medicine, The Catholic University of Korea, Seoul, Republic of Korea; 2 Department of Applied Statistics, Yonsei University, Seoul, Republic of Korea; 3 Division of Pulmonary, Allergy and Critical Care Medicine, Department of Internal Medicine, Bucheon St. Mary’s Hospital, College of Medicine, The Catholic University of Korea, Seoul, Republic of Korea; 4 Division of Pulmonary, Allergy and Critical Care Medicine, Department of Internal Medicine, Yeouido St. Mary’s Hospital, College of Medicine, The Catholic University of Korea, Seoul, Republic of Korea; 5 Division of Pulmonary, Allergy and Critical Care Medicine, Department of Internal Medicine, Incheon St. Mary’s Hospital, College of Medicine, The Catholic University of Korea, Seoul, Republic of Korea; 6 Division of Pulmonary, Allergy and Critical Care Medicine, Department of Internal Medicine, Uijeongbu St. Mary’s Hospital, College of Medicine, The Catholic University of Korea, Seoul, Republic of Korea; 7 Division of Pulmonary, Allergy and Critical Care Medicine, Department of Internal Medicine, Seoul St. Mary’s Hospital, College of Medicine, The Catholic University of Korea, Seoul, Republic of Korea; 8 The Cancer Research Institute, College of Medicine, The Catholic University of Korea, Seoul, Republic of Korea; 9 Division of Pulmonary, Critical Care and Sleep Medicine, Department of Internal Medicine, St. Vincent’s Hospital, College of Medicine, The Catholic University of Korea, Seoul, Republic of Korea

**Keywords:** Non-small cell lung cancer, Cigarette smoking, Screening

## Abstract

**Background:**

Screening for early detection of lung cancer has been performed in high-risk individuals with smoking history. However, researches on the distribution, clinical characteristics, and prognosis of these high-risk individuals in an actual cohort are lacking. Thus, the objective of this study was to retrospectively review characteristics and prognosis of patients with smoking history in an actual lung cancer cohort.

**Methods:**

The present study used the lung cancer cohort of the Catholic Medical Centers at the Catholic University of Korea from 2014 to 2017. Patients with non-small cell lung cancer were enrolled. They were categorized into high and low-risk groups based on their smoking history using the national lung screening trial guideline. Distribution, clinical characteristics, and survival data of each group were estimated.

**Results:**

Of 439 patients, 223 (50.8%) patients were in the high-risk group. Patients in the high-risk group had unfavorable clinical characteristics and tumor biologic features. Overall survival of the high-risk group was significantly shorter than that of the low-risk group with both early (I, II) and advanced stages (III, IV). In multivariate analysis, heavy smoking remained one of the most important poor clinical prognostic factors in patients with lung cancer. It showed a dose-dependent relationship with patients’ survival.

**Conclusions:**

High-risk individuals had poor clinical outcomes. Patients’ prognosis seemed to be deteriorated as smoking amount increased. Therefore, active screening and clinical attention are needed for high-risk individuals.

## Background

Although the therapeutic paradigm of lung cancer has changed drastically due to the appearance of target and immunotherapy, advanced lung cancer still has high mortality rate and poor prognosis [1]. In addition, because treatment cost for lung cancer is enormous, economic burden has been one of major social problems [2]. Therefore, several countries have been trying to provide national screening program for early detection of lung cancer for many years. The most representative one is the National Lung Screening Trial (NLST) in the United States (US). It has been conducting low-dose computed tomography (LDCT) for high risk individuals annually for 3 years, showing an increase of early-stage lung cancers diagnosed and 20% of relative reduction in mortality from lung cancer [3–5]. After this trial, the Centers for Medicare & Medicaid Services (CMS) in the US decided to provide lung cancer screening program to participants aged from 55 to 77 years with heavy smoking (at least 30 pack-years of exposure and with smoke exposure within 15 years) [6]. Another large population-based lung cancer screening trial, the NELSON trial, showed similar results [7]. In this trial, individuals who had smoked at least 15 cigarettes daily for 25 years or 10 cigarettes daily for 30 years and were still smoking or stopped smoking less than 10 years ago were enrolled. Among asymptomatic men at high risk for lung cancer, CT screening led to 26% reduction in lung cancer deaths at 10 years of follow up. However, there could be subtle differences in the standard of eligible smoking amount among countries and their screening programs, depending on health care policy and economic status of each country. Until recently, many studies on effects of smoking on lung cancer prognosis have been published [8, 9], however, they focused on a specific group, such as patients underwent surgery or they had insufficient data for accurate smoking amount of study population. Also, there has been little research on the distribution and clinical features of actual lung cancer population who meet the screening criteria.

Therefore, in this study, we classified patients in an actual lung cancer cohort according to their smoking history based on the NLST eligible criteria. The objective of this study was to retrospectively review characteristics and prognosis of these patients according to smoking amount.

## Methods

### Data source

Since October 2014, seven hospitals of the Catholic University of Korea have consecutively enrolled lung cancer patients. The seven university hospitals are Seoul St. Mary’s Hospital, Yeouido St. Mary’s Hospital, Eunpyeong St. Mary’s Hospital, Uijeongbu St. Mary’s Hospital, Bucheon St. Mary’s Hospital, Incheon St. Mary’s Hospital, and St. Vincent’s Hospital. The Catholic Medical Center (CMC) lung cancer registry was formed by these seven university hospitals. The CMC lung cancer registry registered patients’ symptoms, comorbid diseases, quality of life, and smoking history (smoking status, amounts, and cessation duration) through a same questionnaire at the time of lung cancer diagnosis (Supplementary 1). Smoking status was defined as our previous work [10, 11]. Current smoker was defined a patient who continued smoking upon diagnosis or stopped smoking less than 1 month before diagnosis of lung cancer. Ex-smoker was defined as a patient who had stopped smoking at least 1 month before the diagnosis. Never smoker was defined a patient who had never smoked or had smoked fewer than 100 cigarettes in their lifetime. Pulmonary function tests were carried out following the American Thoracic Society/European Respiratory Society standardization guidelines. By qualified data managers, clinical information including stage, pathology, treatment modality, and survival were systematically recorded to improve the accuracy of data. Researchers were permitted to conduct this study by accessing dataset newly assigned with a serial number whose personal information was removed. This study was approved by the Clinical Research Ethics Committee of the Catholic Medical Center (approval number: XC140IMI0070).

### Study population

**Fig. 1:**
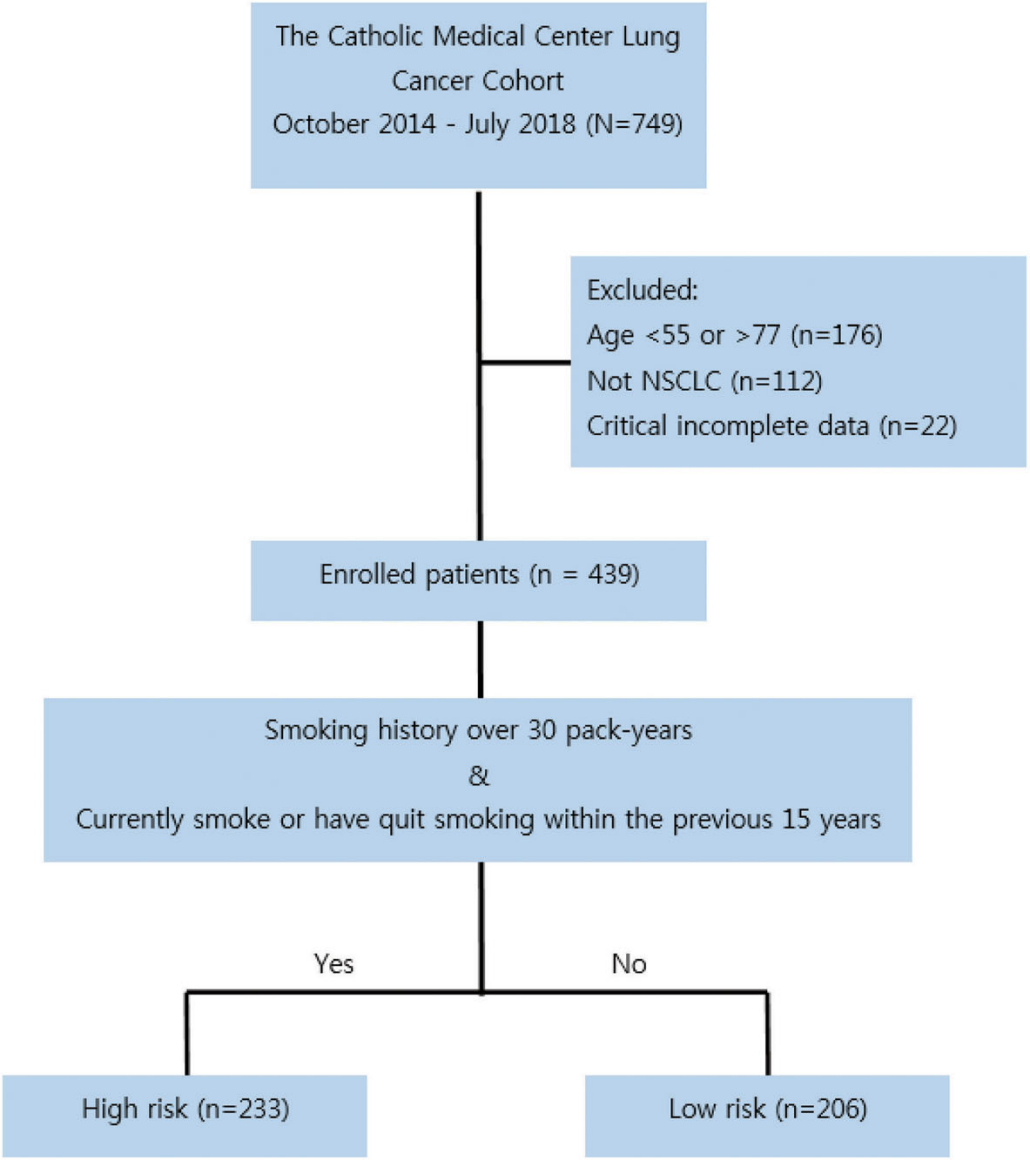
Study flow diagram

We screened 749 patients newly diagnosed with primary lung cancer registered in the CMC lung cancer registry from October 2014 to July 2018. Patients with small cell lung cancer and those aged below 55 years or over 77 years were excluded. Those with incomplete smoking history or clinicopathological data were also excluded. A total of 439 patients were enrolled in this study. They were divided into two groups according to their smoking status and lifetime smoking exposure. Patients with a smoking history of over 30 pack-years and former smokers with less than 15 years after smoking cessation were classified as high-risk group. Others were classified as low-risk group based on the NLST eligible criteria (Fig. 1).

### Data collection

We retrospectively reviewed and collected clinical information from the database of the CMC lung cancer registry. Initial symptoms and quality of life through questionnaire survey, clinical characteristics including age, sex, body mass index (BMI), smoking history, comorbidities, stage by tumor-node-metastasis stage (the 7th edition of the AJCC TNM staging system), histological and cytological diagnosis, pulmonary function results, treatment modalities, and treatment outcomes were collected prospectively and systematically. For detecting and genotyping of epidermal growth factor receptor (EGFR) mutation, peptide nucleic acid (PNA)-mediated PCR clamping method was used with PNAClamp™ EGFR Mutation Detection Kit (PANAGENE, Inc., Daejeon, Korea) [12]. Anaplastic lymphoma kinase (ALK)-fluorescence in situ hybridization (FISH) was performed using Vysis LSI ALK Dual Color Break Apart Probe (Abbott Molecular, Abbott Park, IL, USA), a break-apart probe specific to the ALK locus. Overall survival (OS) was defined as the time from the date of diagnosis to death.

### Statistical analysis

To analyze clinical characteristics between the two groups, two sample t-test was used for continuous variables and Chi-square test was used for categorical variables. Overall survival (OS) was estimated from Kaplan-Meier survival curves. Log-Rank tests were utilized to determine if risk groups were statistically different from each other. Cox proportional hazards model was used for seeking independent prognostic factors in the study. Optimal cutoff points for the smoking pack-years which distinguish patients’ survival were determined by the time-dependent receiver operating characteristic (ROC) curve [13]. As the obtained cutoff points, patients were divided into subgroups according to those, and the Kaplan-Meier survival curves were calculated. A *p* value < 0.05 was considered significant. All analyses were performed using R (version 3.5.1; R Computing, Vienna, Austria).

## Results

### Patients’ characteristics

According to risk stratification by smoking, 223 (53.1%) patients were in the high-risk group and 206 (46.9%) patients were in the low-risk group. Clinical characteristics of each group are shown in Table 1. The percentage of males and the proportion of current smoker were higher in the high-risk group (97.0% vs. 56.3%, *p* < 0.001; 57.1% vs. 16.0%, *p* < 0.001, respectively). The average smoking pack-years and abstinence duration were 55.0 and 5.3 years in the high-risk group, and 11.2 and 15.1 years in the low-risk group, respectively. The cumulative smoking exposure was significantly lower in the low risk group. There were no significant differences between the two groups in terms of comorbidities such as tuberculosis, diabetes, heart disease, or other cancer histories affecting outcome. Overall pulmonary functions including forced vital capacity (FVC) (81.7% vs. 87.9%, *p* = 0.001), forced expiratory volume in 1 s (FEV1) (72.9% vs. 88.5%, *p* < 0.001), FEV1 / FVC ratio (0.63 vs. 0.72, *p* < 0.001), and diffusion capacity of the lung for carbon monoxide (74.3% vs. 82.7%, *p* < 0.001) was significantly lower in the high-risk group. The proportion of patients with advanced stages (III, IV) that were difficult to perform surgery was higher in the high-risk group (III: 36.9% vs. 12.1%, *p* < 0.001; IV: 40.3% vs. 33.5%, *p* < 0.001). Histologically, squamous cell type and poor differentiation were more predominant in the high-risk group compared to those in the low-risk (56.2% vs. 18.9%, *p* < 0.001; 27.9% vs. 19.4%, *p* = 0.001). In the high-risk group, the proportion of patients with EGFR mutations was significantly smaller than that in the low-risk group (11.8% vs. 39.1%, *p* < 0.001). Subtypes of 19 del (4.8% vs. 21.2%, *p* < 0.001) and L858R (4.8% vs. 13.6%, *p* = 0.006) also had smaller proportions in the high-risk group. The proportion of patients who underwent surgery was significantly lower in the high-risk group (37.3% vs. 49.0%, *p* = 0.018). However, the proportion of patients who received chemotherapy or radiotherapy did not show any statistical significance between the two groups (chemotherapy: 67.0% vs. 63.6%, *p* = 0.052; radiation: 25.8% vs. 25.2%, *p* = 0.990).

**Table 1:** Patient characteristics

GROUP	High (*N* = 233)	Low (*N* = 206)	*p*
Age	67.3 ± 6.2	67.1 ± 6.4	0.670
Sex			0.000
Male	226 (97.0%)	116 (56.3%)	
Female	7 (3.0%)	90 (43.7%)	
BMI	22.6 ± 3.1	23.4 ± 2.8	0.003
Symptoms at presentation	143 (61.4%)	96 (46.6%)	0.003
Smoking status			0.000
Current	133 (57.1%)	33 (16.0%)	
Ex + Never	100 (42.9%)	173 (84.0%)	
Pack-years	55.0 ± 19.5	11.2 ± 14.1	0.000
Abstinence duration	5.3 ± 4.8	15.1 ± 12.1	0.000
Comorbidities			
Tuberculosis	46 (19.7%)	31 (15.0%)	0.244
Diabetes mellitus	77 (33.0%)	52 (25.2%)	0.092
Heart disease	38 (16.3%)	25 (12.1%)	0.268
Other cancer history	32 (13.7%)	30 (14.6%)	0.911
Cancer			
Stomach cancer	5 (2.1%)	6 (2.9%)	0.836
Colon cancer	4 (1.7%)	6 (2.9%)	0.605
Thyroid cancer	2 (0.9%)	6 (2.9%)	0.212
Hepatoma cancer	4 (1.7%)	3 (1.5%)	1
Renal cell cancer	4 (1.7%)	2 (1.0%)	0.795
Bladder cancer	3 (1.3%)	2 (1.0%)	1
Pancreatic cancer	1 (0.4%)	1 (0.5%)	1
Uterine cervix cancer	1 (0.4%)	2 (1.0%)	0.915
Biliary cancer	0 (0.0%)	1 (0.5%)	0.951
Ovary cancer	0 (0.0%)	1 (0.5%)	0.951
Prostate cancer	2 (0.9%)	0 (0.0%)	0.533
Breast cancer	1 (0.4%)	1 (0.5%)	1
Rectal cancer	3 (1.3%)	1 (0.5%)	0.704
other cancer	6 (2.6%)	4 (1.9%)	0.902
Clinical Stage			0.000
I/II	69 (29.6%)	98 (47.6%)	
III	86 (36.9%)	25 (12.1%)	
IV	78 (33.5%)	83 (40.3%)	
Histology			0.000
Adeno	94 (40.3%)	156 (75.7%)	
Squamous	131 (56.2%)	39 (18.9%)	
Large	1 (0.4%)	0 (0.0%)	
Other	7 (3.0%)	11 (5.3%)	
Differentiation			0.001
Well	13 (5.6%)	34 (16.5%)	
Moderate	87 (37.3%)	84 (40.8%)	
Poorly	65 (27.9%)	40 (19.4%)	
Unknown	68 (29.2%)	48 (23.3%)	
Driver mutation			
EGFR	22 (11.8%)	72 (39.1%)	0.000
19Del	9 (4.8%)	39 (21.2%)	0.000
L858R	9 (4.8%)	25 (13.6%)	0.006
Others	4 (2.2%)	8 (4.3%)	0.368
ALK	7 (3.9%)	5 (2.8%)	0.769
Pulmonary function			
FVC(%)	81.7 ± 18.0	87.9 ± 20.7	0.001
FEV1(%)	72.9 ± 21.4	88.5 ± 22.5	0.000
FEV1/FVC	0.63 ± 0.13	0.72 ± 0.10	0.000
DLCO(%)	74.3 ± 20.8	82.7 ± 20.6	0.000
Treatment			
Surgery	87 (37.3%)	101 (49.0%)	0.018
Chemotherapy	156 (67.0%)	131 (63.6%)	0.523
Mean cycle	3.6 ± 2.0	4.0 ± 2.3	0.139
Mean line	1.5 ± 0.5	1.4 ± 0.5	0.059
Radiation	60 (25.8%)	52 (25.2%)	0.990

Values are presented as mean ± standard deviation or number (%)

*BMI* body mass index, *EGRF* Epidermal growth factor receptor, *ALK* Anaplastic lymphoma kinase, *FVC* forced vital capacity, *FEV1* forced expiratory volume in 1 s, *FEV1/FVC* forced expiratory ratio, *DLCO* diffusion capacity of the lung for carbon monoxide

### Overall Survival (OS)

**Fig. 2:**
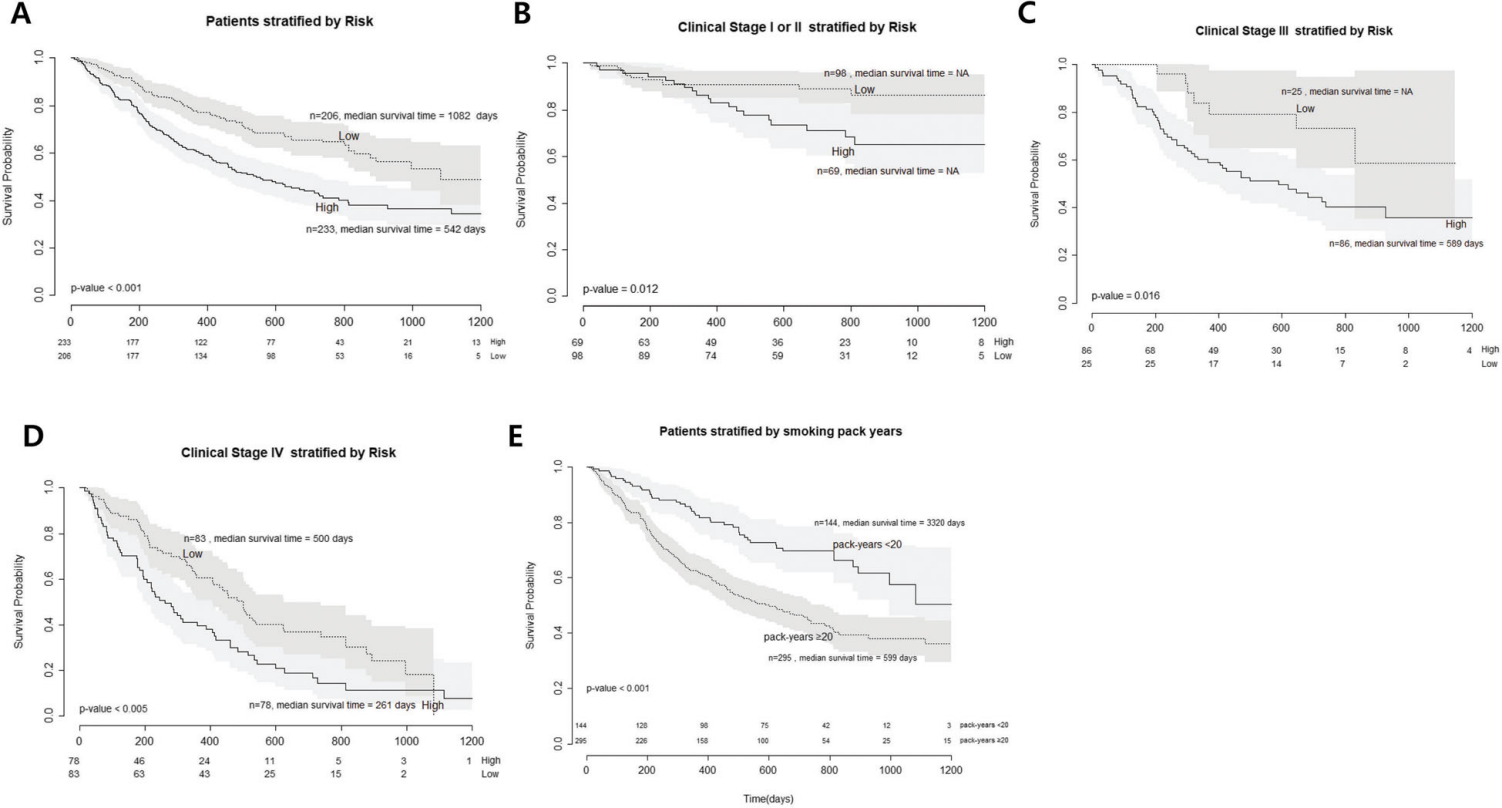
Kaplan-Meier estimate of overall survival of subjects stratified by risk and stage Panel **a** shows the difference of overall survival for patients with high and low risk and Panel **b, c, d** show the difference of overall survival for patients with high and low risk group according to cancer stage. Panel **e** shows the difference of overall survival according to MILD trial eligible criteria

OS was found to be statistically lower in the high-risk group. Median survival time of high-risk and low-risk groups were 542 and 1082 days, respectively (*p* < 0.001) (Fig. 2a). When patients were divided into stage I-II, III, and IV to compare the OS according to stages, the high-risk group had significantly poorer OS in all stages (Fig. 2b~d). We additionally performed survival analysis using the Multicentric Italian Lung Detection (MILD) trial eligible criteria (minimum of 20 pack-years smoking history). Results showed heavy smoker had poorer OS compared to light or never smoker group (*p* < 0.001) (Fig. 2e).

### Independent factors associated with overall survival

**Fig. 3:**
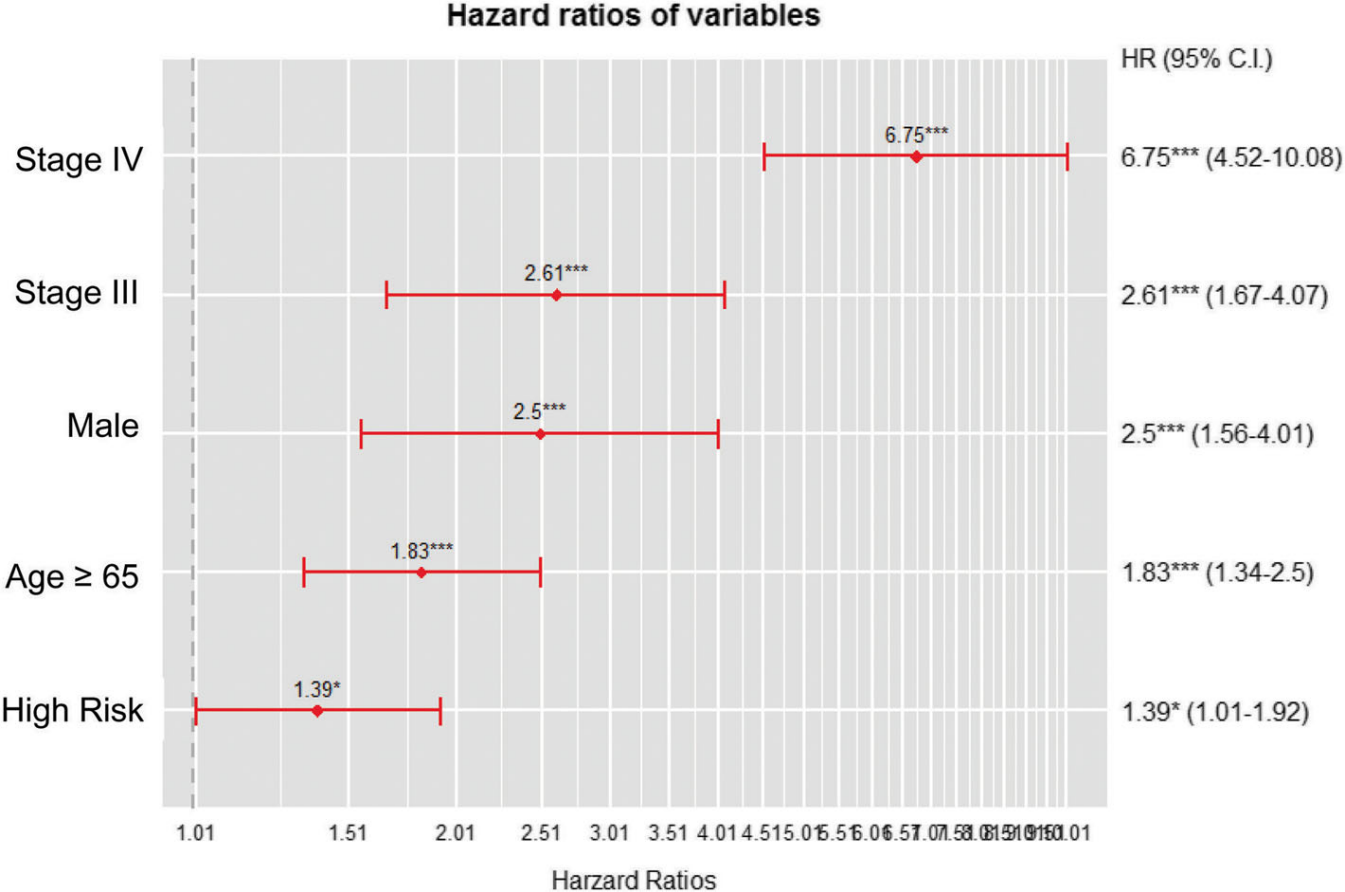
Cox Proportional hazards model for overall survival

Multivariate Cox proportional hazards model was used to identify factors independently associated with the OS of lung cancer patients. Advanced stage (IV) [hazard ratio (HR), 6.75; 95% confidence interval (CI): 4.518–10.082], male (HR, 2.50; 95% CI: 1.558–4.01), old age (HR, 1.83; 95% CI: 1.339–2.502), and heavy smoking (HR, 1.39; 95% CI: 1.006–1.923) were independently associated with poor survival outcome (Fig. 3).

### Prognosis according to amount of lifetime cigarette smoking

**Fig. 4:**
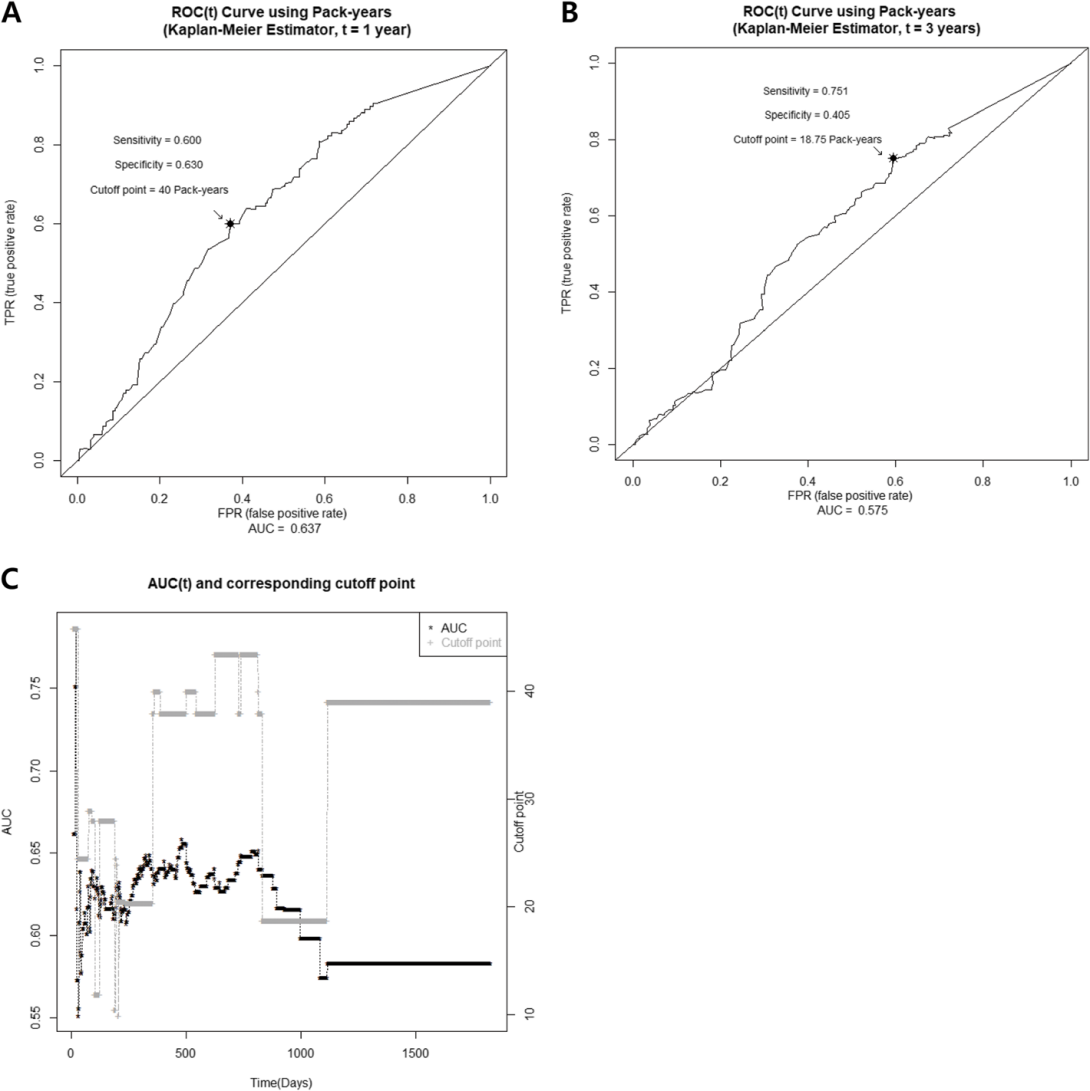
The optimal cutoff points to distinguish patients’ survival on time-dependent ROC curve. Panel **a** and **b** shows the ROC curve for 1 and 3-year mortality, respectively. Panel **c** shows the time-dependent AUC and corresponding cutoff points through the whole study period

**Fig. 5:**
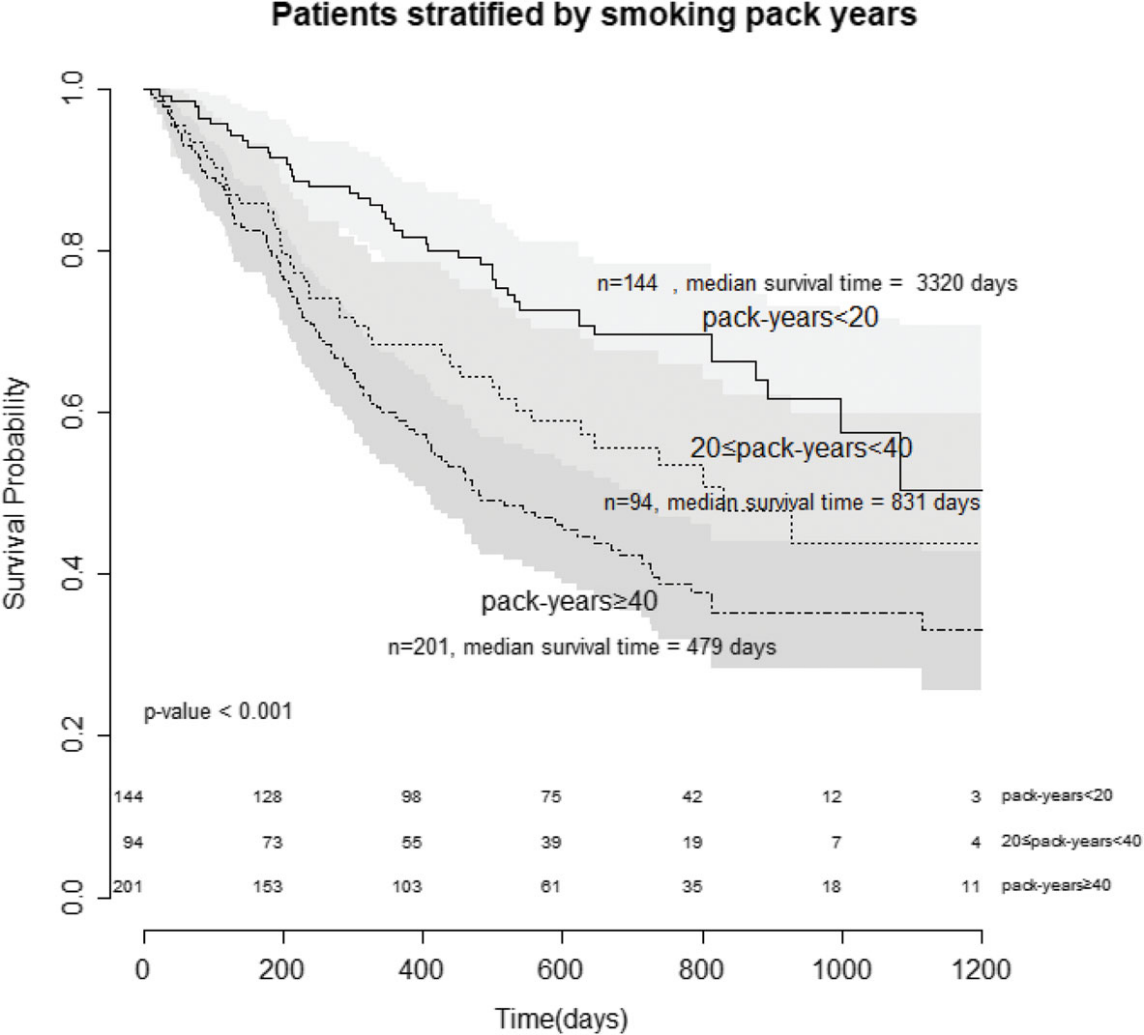
Kaplan-Meier estimate of overall survival of subjects stratified by amount of lifetime cigarette smoking

To assess the impact of the amount of lifetime cigarette smoking on prognosis of lung cancer, additional survival analysis was performed. Using the time-dependent ROC curve, it’s shown that 40 pack-years (Fig. 4a) and 18.75 pack-years (Fig. 4b) were the optimal cutoff points which distinguish between patients who died by one and 3 years and those who did not, respectively. Sensitivity, specificity, and AUC scores for cutoff points 40 pack-years to 1 year mortality and 18.75 pack-years to 3 year mortality were: 0.60, 0.63, 0.637 and 0.751, 0.405, 0.575. Time-dependent AUC and corresponding cutoff points through the whole study period were showed in Fig. 4c. According to the obtained cutoff points, patients were divided into three groups: over 40 pack-years, 20–40 pack-years, and below 20 pack-years. As the smoking amount increased, patients’ survival was found to be worse stepwise, showing statistically significant difference (median survival time of below 20 pack-years vs. 20–40 pack-years vs. over 40 pack-years: 3320 vs. 831 vs. 479 days, *p* < 0.001) (Fig. 5).

## Discussion

The purpose of this study was to analyze the effect of smoking on clinical characteristics and outcomes of patients with lung cancer, especially NSCLC, by dividing patients according to the NLST eligible criteria. In the present study, the high-risk group who met the NLST criteria were associated with poor prognostic factors such as male, advanced stage, low BMI, squamous cell type and poor differentiation in histology, lower driver mutation, and poor pulmonary functions compared with the low-risk group. Clinical outcomes represented by OS were also poorer for the high-risk group than those for the low-risk. This study revealed that smoking was an independent factor associated with the prognosis of lung cancer, showing a dose-dependent relationship.

Previous studies have shown that smokers among lung cancer patients have more airflow limitation than never smokers. They also tend to have lower BMI and poorer clinical outcome [14]. It has also been reported that smokers have more advanced stage and worse histologic types such as small cell or squamous cell [15–17]. Results of the present study were consistent with previous results. There are several reasons why the high-risk group has poor prognosis. More exposure to smoking causes more oxidative stress at cell level, leading to greater genetic damage. Smoking causes transformation of normal cells into cancer cells with large mutation burden and poor histologic type [18–20]. Also, it induces chronic airway and systemic inflammation, leading to impaired lung function and sarcopenia that are closely related to poor prognosis in lung cancer [21, 22]. In this study, even after adjusting for cancer stage which had the most significant effect on survival, the OS of the high-risk group was significantly decreased in both early (I, II) and advanced stages (III, IV). Previous studies have reported that poor treatment outcomes of early lung cancer patients in smokers are related to early recurrences [23, 24]. On the other hand, in patients with advanced lung cancer, patients’ comorbidities and intolerance to the treatment are important factors associated with poor prognosis [25]. However, in this study, there were no significant differences in comorbidities or the number of chemotherapies between high-risk and low-risk groups. O’Malley M et al. have insisted that smoking can negatively impact drug pharmacokinetics and efficacy during chemotherapy in advanced lung cancer. In addition, smoking may result in suboptimal therapy and development of drug resistance [26]. Therefore, there is a need to pay more attention to the risk of smoking itself.

In multivariate Cox proportional hazards model, advanced stage, old age, and male patients had worse outcomes. Importantly, even after adjusting for major prognostic factors such as stage, sex, age, smoking remained significant prognostic factor in lung cancer. To further clarify the effect of smoking on the prognosis of lung cancer, we additionally carried out analysis about the impact of smoking amount on mortality. As smoking amount increased, OS was statistically significantly deteriorated. Smoking had a dose-dependent relationship with patients’ survival. We could draw conclusion that smoking is a strong contributor to the poor prognosis of lung cancer patients. Therefore, lung cancer screening using LDCT in heavy smokers is very important. In actual clinical practice, however, the participation rate of screening program remains at about 50 to 60% and participants are mainly older patients [27, 28]. Therefore, in recent years, interest in disparity of lung cancer screening and efforts to solve it have been made [29, 30]. One of them is the expansion of screening eligibility according to smoking amount. In some countries such as Italy and Denmark where the proportion of smokers and lung cancer mortality are high, trials have been conducted to expand lung cancer screening participants, loosening the standard of smoking amount to 20 pack-years [31, 32]. Especially, in the MILD trial, employing these criteria (minimum of 20 pack-years smoking history), the reduction of lung cancer mortality was greater compared with the NLST trial [31]. In our study, according to survival analysis using MILD trial eligible criteria, there was a significant survival difference between the two groups. Therefore, the necessity of expanding the target population of lung cancer screening is also emerging. In the present study, significant mortality differences were observed even between 20 and 40 pack-years and below 20 pack-years. Additional study is needed to extend lung cancer screening criteria in the future.

In addition to screening lung cancer early, it is also necessary to try interventions for active smoking cessation for screening subjects with current smoking. Studies have shown that smoking at the time of lung cancer diagnosis is a major prognostic factor in lung cancer. There were evidences that active smoking cessation interventions can improve the prognosis of lung cancer [24, 33]. In this study, current smokers accounted for 38.5% of all patients (57.1% in the high-risk group). It seemed that smoking cessation intervention should be emphasized when managing these patients, especially for those in the high-risk group. Recently, a national effort has been made to link smoking-cessation service with lung cancer screening [34].

This study has some limitations. First, we only analyzed patients with specific ages and NSCLC, not all lung cancer patients. Patients with small cell lung cancer were excluded because their treatment morbidity and prognosis could be significantly different. Second, our study showed a relatively low specificity for cutoff points 18.75 pack-years to 3-year mortality in the ROC curve for the impact of the smoking amount on prognosis of lung cancer. Because this is an analysis according to the smoking amount, not the presence or absence of smoking, we think that the specificity of concretely quantified smoking amount for lung cancer mortality may be relatively low. However, as shown in Fig. 4c, the AUC value was maintained over 0.5 during the entire study period. In addition, since the AUC values of 1-year and 3-year mortality are 0.637 and 0.575, we do not believe that our model shows negative result in predicting survival according to the smoking amount in lung cancer. Also, the sensitivity and specificity can be influenced by sample size. Therefore, further prospective studies with larger sample size are needed for better predictability and external validation of our model. Third, this study was performed in a retrospective manner. However, medical records and patients’ questionnaire were faithfully collected from the time of enrollment and data were re-examined by authorized data managers. Therefore, data including smoking history, clinical characteristics, and clinical outcome were high qualified and reliable. Moreover, this registry covered patients with lung cancer in seven hospitals in the Republic of Korea. They could represent lung cancer patients in Korea general population to some extent.

## Conclusion

High-risk individuals with long-term and heavy smoking who met the NLST eligible criteria were found to have poor prognosis. Also, as the degree of smoking amount increased, the prognosis of patients deteriorated. Therefore, it is necessary to recommend more active screening to heavy smokers and give more clinical attention to them to improve their clinical outcomes. Additional research on the need of expanding lung cancer screening to intermediate-risk group is needed in the future.

## Supplementary information

**Additional file 1.** Questionnaire used when collecting data in this study.

## Data Availability

The dataset used and analyzed during the present study is available from the corresponding author upon reasonable request.

## Abbreviations

NSCLC: Non-small cell lung cancer
NLST: The national lung screening trial
US: The United States
LDCT: Low-dose computed tomography
CMS: The Centers for Medicare & Medicaid Services
CT: Computed tomography
BMI: Body mass index
EGFR: Epidermal growth factor receptor
PNA: Peptide nucleic acid
ALK: Anaplastic lymphoma kinase
FISH: Fluorescence in situ hybridization
OS: Overall survival
FVC: Forced vital capacity
FEV1: Forced expiratory volume in 1 s
DLCO: Diffusion capacity of the lung for carbon monoxide
MILD: The Multicentric Italian Lung Detection
HR: Hazard ratio
CI: Confidence interval
