# Supplementary material for: Impact of smoking amount on clinicopathological features and survival in non-small cell lung cancer
Source: BMC Cancer. 2020 Sep 3;20:848. doi: 10.1186/s12885-020-07358-3 (PMC7469911; doi:10.1186/s12885-020-07358-3)
Supplement: Supplementary file 1 — Additional file 1. Questionnaire used when collecting data in this study. [file 12885_2020_7358_MOESM1_ESM.docx]

**=** **Questionnaire =**

This questionnaire is intended to help you accurately determine your medical history.

If you fill out in detail, it can be very helpful for proper management.

Date : YYYY/MM/DD

Height : ______ cm Weight : ______ kg

**1. Cause of visit**

□ Complaint of symptoms

□ Abnormal finding on the chest image

**2. The following are questions about your respiratory symptoms. Please mark each item in the appropriate box.**

1) Cough □ Yes □ No

2) Pleghm □ Yes □ No

3) Chest pain □ Yes □ No

4) Hoarseness □ Yes □ No

5) Wheezing or crackle □ Yes □ No

6) Dyspnea

**□ 0 point**: I only get breathless with strenuous exercise

**□ 1 point**: I get short of breath when hurrying on level ground or walking up a slight hill

**□ 2 point**: On level ground, I walk slower than people of the same age because of breathlessness, or I have to stop for breath when walking at my own pace on the level

**□ 3 point**: I stop for breath after walking about 100 yards or after a few minutes on level ground

**□ 4 point**: I am too breathless to leave the house or I am breathless when dressing

7) Hemoptysis □ Yes □ No

Please check only if you have hemoptysis.

□ It's about blood tinged on the phlegm.

□ The amount of hemoptysis is higher than ______ cc a day.**3. The following are questions about your systemic symptoms. Please mark each item in the appropriate box.**

1) Weight loss □ Yes □ No

※ If there was weight loss, how much was the weight loss in kg over 6 months?

2) Fatigue □ Yes □ No

3) General weakness □ Yes □ No

4) Loss of appetite □ Yes □ No

5) Pain (Other areas except chest) □ Yes □ No

※ Specific areas of pain :

**If you have chest pain, please check 2. Respiratory symptoms 3) Chest pain.**

6) Febrile sense □ Yes □ No

**4. The following are questions about your smoking history. Please mark each item in the appropriate box.**

1) Have you ever smoked?

□ Yes

□ No (If you have smoked less than 5 packs in your lifetime, please mark ‘No’.)

※ Only those who answered 'yes', please answer the following questions.

2) When was the first time you smoked?

___ years

3) How much did you smoke a day during the smoking period?

___ pack a day

4) Do you still smoke?

□ Yes

□ No

※ Only those who answered ‘no’, please answer question 5).

5) When did you stop smoking?

___ years

6) Excluding the last smoking cessation period, how much was the total amount of smoking period before?

(Please answer both current and past smokers.)

□ I never tried to quit smoking before

□ I have tried to quit smoking before (Duration of smoking cession: ___________)

**5. The following are questions about comorbid diseases. Please mark each item in the appropriate box.**

(※ Please fill out all the medical conditions you are suffering from in the past or present)

**※ Respiratory diseases**

1) Tuberculosis □ Yes □ No

※ If you suffered tuberculosis, when were you diagnosed? ________ years

Disease status : Cured/Currently on treatment/Denial

2) COPD □ Yes □ No

3) Asthma □ Yes □ No

4) ILD □ Yes □ No

5) Silicosis □ Yes □ No

6) Asbestosis □ Yes □ No

**※ Other diseases**

1) Heart disease □ Yes □ No

2) Hypertension □ Yes □ No

3) Diabetes □ Yes □ No

4) Other diseases ( )

**6. The following are questions about comorbid malignancy.**

**Have you ever been diagnosed with cancer in the past or present?**

□ Yes

□ No

※ Only those who answered ‘yes’, please describe the disease status in detail.

(Excluding lung cancer or metastatic cancer caused by lung cancer)

________________________________________________________________
